# Posterior Microphthalmos Pigmentary Retinopathy Syndrome

**DOI:** 10.18502/jovr.v18i2.13190

**Published:** 2023-04-19

**Authors:** Syed Saifuddin Adeel, Syed Mohideen KA, Anuj Sharma, Vinit J Shah, Adheesh Shetty

**Affiliations:** ^1^Department of Retina Vitreous, Aravind Eye Hospital, Tirunelveli, Tamil Nadu, India; ^2^Retina Services, Neoretina Eyecare Institute, Hyderabad, Telangana, India

**Keywords:** Foveoschisis, MFRP Gene, Microphthalmos, Posterior Microphthalmos, Retinitis Pigmentosa

## Abstract

**Purpose:**

To report a case of a rare disease entity Posterior Microphthalmos Pigmentary Retinopathy Syndrome (PMPRS) in a 47-year-old female with a brief review of literature.

**Case Report:**

A 47-year-old woman presented with a history of defective vision with an associated difficulty in night vision. Clinical workup was done, which included a thorough ocular examination showing diffuse pigmentary mottling of fundus, ocular biometry showing short axial length with normal anterior segment dimensions, electroretinography showing extinguished response, optical coherence tomography showing foveoschisis, and ultrasonography showing thickened sclera–choroidal complex. Findings were consistent with those reported by other authors with PMPRS.

**Conclusion:**

Posterior microphthalmia with or without other ocular and systemic associations should be suspected in cases with high hyperopia. It is mandatory to carefully examine the patient at presentation and close follow-ups are needed to maintain visual function.

##  INTRODUCTION

Microphthalmia is a condition characterized by axial length (AXL) of the eye being less than two standard deviations with the normal for that age.^[1]^ It can be simple, presenting as an isolated entity or complex, associated with other malformations. Furthermore, it can also be sub-classified as nanophthalmos, anterior microphthalmos, and posterior microphthalmos. Nanophthalmos or simple microphthalmos is a condition wherein the AXL is short due to both small anterior and posterior segments.

Posterior microphthalmia (PM), first termed by Spitznas et al^[2]^ is a rare entity characterized by shorter AXL with smaller posterior segment dimensions in association with sclero-choroidal thickening and normal anterior segment. Although majority of PM cases have been reported as sporadic, an autosomal recessive form of inheritance is proposed for familial cases. Few case reports have documented an association with uveal effusion syndrome,^[3]^ pigmentary retinopathy, foveoschisis, papillo-macular retinal folds,^[2]^ macular hole,^[4]^ and retinal dialysis^[5]^ amongst other conditions.

A few reports of a new syndrome called Posterior Microphthalmos Pigmentary Retinopathy Syndrome (PMPRS) characterized by PM, foveoschisis, retinitis pigmentosa, and optic disc drusen have been recently described. Literature on this new entity is however sparse with the largest reported case series of five family members by Morillo Sánchez et al^[6]^ in 2019. Mukhopadhyay et al^[7]^ in 2010 described three novel mutations in the gene related to this syndrome in seven individuals from four families; they called the ocular condition as Membrane type Frizzled-Related Protein (*MFRP*)-related oculopathy.

**Table 1:** Ocular biometric measurements of both eyes of the patient.


**Eye**	**Axial length (mm)**	**White to White (mm)**	**Anterior chamber depth (mm)**
Right	18.37	12.00	3.52
Left	18.00	12.40	3.36
	
	
mm, millimeter			

**Figure 1:**
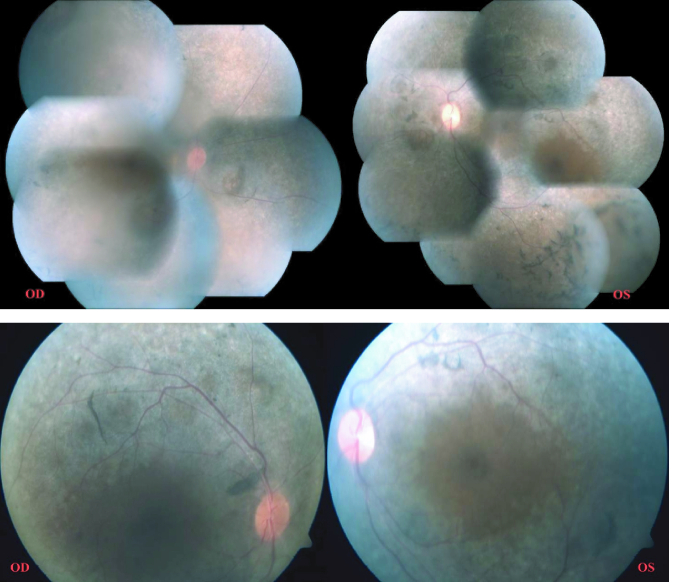
Fundus photograph of the posterior pole of both eyes showing disc pallor, more appreciated in the left eye, pigmentary mottling, and vascular attenuation. **(b) **Fundus montage of both eyes showing pigment clumps and bony spicule pigmentation in mid-peripheral and peripheral retina.

**Figure 2:**
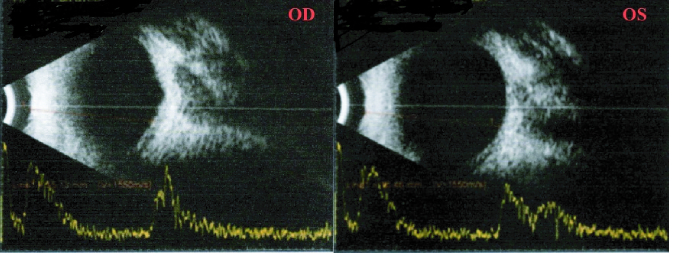
Ultrasound B-scan of both the eyes showing a short axial length with associated sclero-choroidal thickening in the para-papillary area.

**Figure 3:**
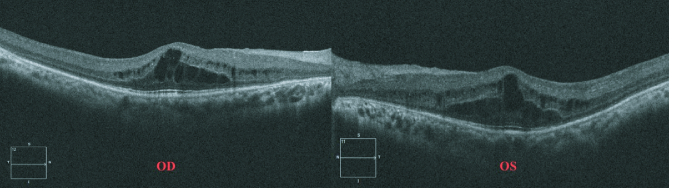
Optical coherence tomography of macula passing through the fovea shows evidence of multiple cystoid elevations in the inner retinal layers suggestive of foveoschisis.

**Figure 4:**
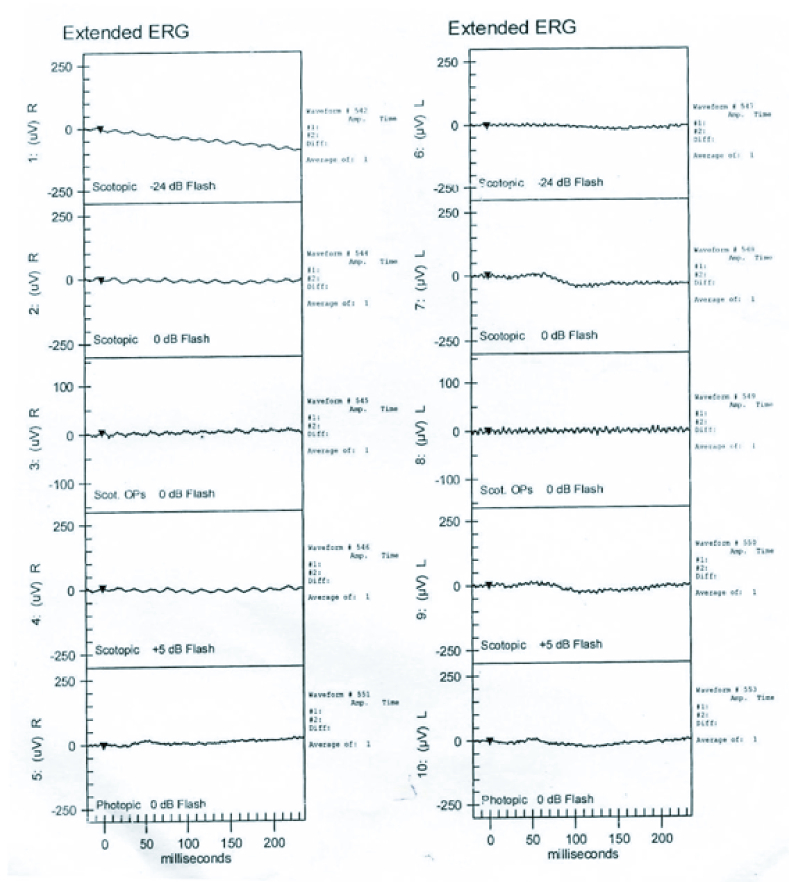
Electroretinogram of the patient shows evidence of extinguished wave response in both eyes.

##  CASE REPORT

A 47-year-old female presented with a history of defective vision in both eyes for many years with an associated difficulty in night vision. She also had a history of using thick glasses since childhood with unremarkable past medical history. She was born from a non-consanguineous marriage and had no systemic illness at presentation. There was no history of any ocular trauma, ocular surgery, or a history of visual dysfunction in her family.

On ocular examination, the visual acuity in the right eye (OD) was 0.25 and 0.16 in the left eye (OS) with a subjective cycloplegic refraction of +10 D. Intra-ocular pressure was 13 mmHg in OD and 12 mmHg in OS as measured by Goldmann Applanation Tonometry. On Slit-lamp examination, her anterior segment was unremarkable with a normal anterior chamber depth (ACD). Vitreous evaluation showed presence of pigmented cells in both eyes. Fundus examination revealed diffuse pigmentary changes characterized by pigment clumps and bone spicule pigmentation of mid-peripheral and peripheral retina, waxy pallor of disc, vascular attenuation, and blunting of the macular reflex [Figures 1a & 1b]. Ultrasound B-scan of both eyes was suggestive of a short AXL and sclero-choroidal thickening [Figure 2]. On ocular biometry, the AXL was 18.37 mm in OD and 18.00 mm in OS, the white-to-white (WTW) corneal measurements were 12.0 mm OD and 12.4 mm OS, and the ACD was 3.52 mm OD and 3.36 mm OS [Table 1]. Optical coherence tomography passing through the fovea showed foveoschisis in both the eyes [Figure 3]. Furthermore, ERG showed extinguished wave response in both the eyes [Figure 4]. She was explained the prognosis and was advised low vision aid for rehabilitation. Although PMPRS is a phenotypic diagnosis, we asked the patient for a genetic workup so as to identify the causative gene. However, the patient denied further investigations in this regard as it did not alter her visual prognosis or treatment outcome.

##  DISCUSSION

Buys et al^[8]^ in 1999 were the first to report a 68-year-old male patient with a combination of retinitis pigmentosa, nanophthalmos, and optic nerve head drusen. They hypothesized that the retinal pigmentation was due to chronic serous retinal detachments and choroidal detachments.

Ayala-Ramirez et al^[9]^ in 2006 described in four siblings of a Mexican family the ophthalmic features of retinitis pigmentosa, foveoschisis, posterior microphthalmos, and optic disc drusen and proposed this disease entity as a new oculo-genetic syndrome. They also described clinical criteria for the diagnosis of this syndrome.

In 2008, similar findings were noted by Crespi et al^[10]^ in a Spanish family with three affected brothers. The authors put forth this disease as a distinct autosomal recessive entity caused by a novel frame-shift mutation in the membrane type frizzled-related protein (*MFRP*) gene. Mutations in the 13-exon *MFRP* gene located on chromosome 11q23 encoding a trans membrane protein with 579 amino acid residues was demonstrated to be present^[7,9,10]^ in prior case reports on PMPRS. Predominantly, it is expressed in ciliary epithelium and the retinal pigment epithelium.

Similarly, our patient had PM with normal anterior segment dimensions. The antero–posterior diameter was 18.7 mm (OD)/18.0 mm (OS), with a hyperopia of +10 D (OU). We found optic disc pallor, diffuse pigmentary changes, vascular attenuation, blunting of the macular reflex, and extinguished ERG response meeting the criteria proposed by Ayala Ramirez et al.^[9]^ However, unlike Ayala Ramirezetal, there was no evidence of optic nerve head drusen and papillo-macular folds.

Pehere et al^[11]^ reported two siblings with PMPRS syndrome and postulated autosomal recessive mode of inheritance. No autofluorescence or clinical evidence of optic nerve head drusen was noted. They postulated that posterior microphthalmos and retinitis pigmentosa may be the constant features of the syndrome, while foveoschisis and optic nerve head drusen may exist as variable features of this syndrome.

In a study of four families, phenotypic variability was reported with PMPRS arising due to *MFRP* gene mutations.^[7]^ The authors noted a variable presence of optic nerve head drusen, serous retinal detachments, and foveal cysts. Contrary to reports of Nasser et al,^[12]^ no subretinal drusenoid deposits or craniofacial malformations were noted in our case.

Albar and co-authors^[13]^ reported a case of high hyperopia that was managed with bilateral clear lens extraction and posterior chamber intra-ocular lens implantation. Although a diagnosis of PM was reached, the authors attributed the foveoschisis to be a postoperative cystoid macular edema for which repeated intravitreal injections of anti-VEGF and steroids were administered. It is pertinent to note that a high index of suspicion in, and a knowledge of retinal pathologies that coexist with, posterior microphthalmos may prevent such futile treatments in patient with high hyperopia.

In conclusion, cases with high hyperopia should be suspected with PM which can occur with other ocular and systemic features. Its correct interpretation is important to avoid the misdiagnosis and subjecting the patient to unnecessary investigations and interventions. Amblyopia therapy and close follow-up is important to improve or maintain visual function that may be compromised due to the existing retinal pathologies.

##  Declaration of Patient Consent

The authors certify that they have obtained all appropriate patient consent forms. In the form the patient has given her consent for his images and other clinical information to be reported in the journal. The patient understand that her name and initial will not be published and due efforts will be made to conceal her identity, but anonymity cannot be guaranteed.

##  Financial Support and Sponsorship

None.

##  Conflicts of Interest

None.
